# Electrostatic Effects on the Stability of 2,4,6‐Trifluoromethylidenecyclohexanes, Cyclohexanones, and Cyclohexanethiones: Beyond Nonconventional Hydrogen Bonding

**DOI:** 10.1002/cphc.70619

**Published:** 2026-10-29

**Authors:** Matheus P. Freitas

**Affiliations:** ^1^ Department of Chemistry Institute of Natural Sciences Federal University of Lavras Lavras Minas Gerais Brazil

**Keywords:** C─F bond, electrostatics, hydrogen bonding, hyperconjugation, six‐membered rings

## Abstract

Six‐membered rings provide valuable frameworks for investigating intramolecular interactions, as their axial and equatorial bonds create distinct chemical environments governed by steric, electrostatic, and hyperconjugative effects. Fluorine is an especially suitable probe for such studies because the C─F bond is among the most polar in organic chemistry. In this context, 2,4,6‐trifluoromethylidenecyclohexanes (**1**), cyclohexanones (**2**), and cyclohexanethiones (**3**) were investigated by quantum chemical calculations at the B3LYP/def2‐TZVP level to evaluate the role of unconventional C─F···H─C hydrogen bonding and electrostatic effects on stereoisomer stability. For compounds **1** and **3**, the *eae* stereoisomer is favored not only by unconventional hydrogen bonding but also by stabilizing electrostatic gauche interactions. Interestingly, the C─F···H─C interaction is weaker than in the monofluorinated counterparts because fluorination decreases the acidity of the α‐hydrogens while simultaneously rendering the α‐carbon atoms positively charged, thereby enhancing favorable F···C electrostatic interactions. Although this electrostatic effect is also present in compound **2**, the *eae* stereoisomer is primarily stabilized by *σ*_C─H_ → π*_C═O_ hyperconjugation, since the equatorial α‐fluorine atoms at C2 and C6 experience dipolar repulsion with the C═O bond, diminishing the electrostatic stabilization. These electrostatic interactions persist in a high‐dielectric medium (water), where increased solvent polarity further stabilizes the more polar stereoisomers.

## Graphical abstract

Quantum‐chemical calculations reveal how unconventional C─F···H─C hydrogen bonding, electrostatic interactions, and hyperconjugation collectively govern the conformational preferences of trifluorinated cyclohexane derivatives. Fluorination weakens C─F···H─C hydrogen bonding while enhancing favorable F···C electrostatic interactions. The results demonstrate that the balance between these effects depends strongly on the molecular framework and remains significant even in aqueous solution.

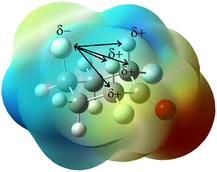

## 1 Introduction

Six‐membered rings provide valuable model systems for investigating intramolecular interactions, as chair interconversion alternates axial and equatorial substituents, thereby exposing them to distinct steric and electronic environments. This feature makes such systems particularly suitable for probing conformational stability. In parallel, organofluorine compounds exhibit unique physicochemical properties largely arising from the highly polarized C─F bond, one of the most polar bonds in organic chemistry [1, 2]. As a consequence, the low‐lying energy of the σ*_C─F_ orbital underlies phenomenon such as the gauche effect [3, 4, 5, 6, 7, 8, 9] while also enabling modulation of molecular lipophilicity [10, 11]. Furthermore, fluorinated organocycles can present polarized faces capable of binding and transporting ions [12, 13, 14], and notably, approximately 25%–30% of pharmaceuticals and agrochemicals contain at least one C─F bond [15, 16, 17, 18].

Given the high polarity of the C─F bond, the partially negative fluorine atom can also participate in unconventional hydrogen bonding interactions with C─H donors, which are predominantly electrostatic in nature [19]. For instance, axial 4‐fluorocyclohexanone is approximately 1.0 kcal mol^−1^ more stable than its ostensibly less sterically hindered equatorial conformer, a preference attributed to favorable syn‐1,3‐diaxial electrostatic interactions [20]. This interpretation, however, contrasts with traditional textbook explanations [21], highlighting the need for further investigation to clarify the role of C─F···H─C interactions in determining the stereochemical stability of fluorinated cyclohexane derivatives.

To address this issue, a series of 2,4,6‐trifluorocyclohexanones and related derivatives, including methylidenecyclohexanes and cyclohexanethiones (Figure 1), were investigated using quantum‐chemical methods. Prof. Juaristi’s group has provided an extensive analysis of related systems, including the role of *n*_F_ → σ*_CX_ (X = H, C, O, S) stereoelectronic interactions in determining conformational preferences [22], the use of the ^1^J_CH_ coupling constant in carbonyl, thiocarbonyl, and methylidene compounds as a probe of stereoelectronic effects on C─H bond lengths [23], and the analysis of related interactions in cyclohexanes, six‐membered heterocycles, and cyclohexanones [24]. Building on these studies, the present systems were selected to assess whether α‐hydrogens can participate in hydrogen‐bonding interactions upon additional fluorine substitution at the C‐2 and C‐6 positions. This question is particularly intriguing because recent studies indicate that geminal fluorination decreases, rather than increases, the electropositive character of hydrogen atoms, despite the expected increase in C─H bond acidity [25, 26]. Calculations were performed both in the gas phase and in implicit aqueous solution, where the stabilization of molecular dipoles is expected to enhance electrostatic interactions.

**FIGURE 1:**
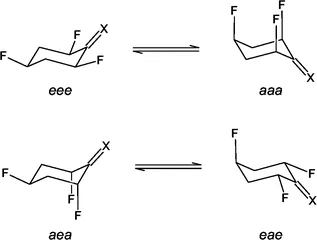
Fluorinated six‐membered rings investigated in this study at the B3LYP/def2‐TZVP level of theory, in both the gas phase and implicit water (X = CH_2_—**1**, O—**2**, and S—**3**).

## 2 Results and Discussion

Two pairs of conformers were evaluated for each derivative, with X = CH_2_ (**1**), O (**2**), and S (**3**). For all compounds, the most stable stereoisomer in the gas phase is the *eae* structure, in which the C2─F and C6─F bonds adopt equatorial orientations, while the C4─F bond is axial (Table 1). This preference is consistent with the previously reported destabilizing effect of dipole–dipole repulsion between 1,3‐diaxial C─F bonds, which can outweigh the stabilizing *n*_F_ → σ*_CX_ geminal stereoelectronic interaction [22]. A consistent trend is also observed in which the *aaa* stereoisomer is the least stable, whereas the *aea* and *eee* forms exhibit similar populations.

**TABLE 1:** Dipole moments (in D), relative standard Gibbs free energies, electronic energies [DLPNO‐CCSD(T)/CBS values in parenthesis], Lewis and non‐Lewis contributions to the electronic energies, and natural Coulombic electrostatic energies for fluorinated six‐membered rings, calculated in the gas phase and in implicit water (SMD), in kcal mol^−1^.

Cpd	Gas						Water					
	***µ***	**Δ*G*** ^**0**^	Δ*E*	***E***_**NL**_	***E***_**L**_	NCE	***µ***	**Δ*G*** ^**0**^	Δ*E*	***E***_**NL**_	***E***_**L**_	NCE
**1** *aaa*	5.2	4.8	5.2 (5.4)	−322.7	327.8	−187.0	7.9	0.7	0.6 (0.7)	−303.1	303.7	−199.9
**1** *eee*	1.5	1.6	1.6 (1.8)	−318.9	320.5	−210.0	2.6	1.3	1.1 (1.0)	−300.9	302.0	−223.7
**1** *aea*	3.4	1.5	1.6 (1.8)	−314.4	316.0	−199.6	5.1	0.0	0.0 (0.0)	−298.3	298.3	−214.1
**1** *eae*	1.8	0.0	0.0 (0.0)	−318.3	318.3	−215.3	2.7	0.3	0.2 (0.1)	−304.0	304.2	−232.0
**2** *aaa*	4.2	5.2	5.4 (5.2)	−349.5	354.9	−225.7	6.4	6.1	6.5 (6.0)	−326.6	333.1	−251.4
**2** *eee*	3.9	2.1	2.1 (2.3)	−362.8	364.9	−215.6	5.9	1.0	1.2 (1.2)	−334.4	335.7	−242.9
**2** *aea*	1.7	2.4	2.7 (2.2)	−341.2	343.8	−235.5	2.7	5.9	6.5 (5.8)	−321.2	327.6	−262.0
**2** *eae*	4.2	0.0	0.0 (0.0)	−361.4	361.4	−223.4	6.0	0.0	0.0 (0.0)	−337.8	337.8	−254.0
**3** *aaa*	4.2	5.1	5.2 (5.0)	−336.9	342.0	−153.4	6.5	5.0	5.1 (4.6)	−319.0	324.0	−165.2
**3** *eee*	3.0	2.1	2.2 (2.3)	−343.9	346.1	−170.0	5.1	1.2	1.2 (1.3)	−324.4	325.6	−173.7
**3** *aea*	1.8	2.3	2.4 (1.9)	−327.8	330.2	−167.6	2.8	4.8	5.0 (4.3)	−313.1	318.1	−180.2
**3** *eae*	3.4	0.0	0.0 (0.0)	−343.5	343.5	−173.8	5.2	0.0	0.0 (0.0)	−327.8	327.8	−181.3

Notably, the energy gap between the *eae* conformer and the other stereoisomers is smaller for compound **1**, indicating that the polarity of the C═X bond plays a significant role in determining conformational stability. Specifically, increasing C═X polarity enhances the relative stabilization of the *eae* conformer compared to the others.

These findings suggest that α‐fluorine substitution could modify the electronic properties of the adjacent hydrogens and, in the *eae* conformer, allow the axial fluorine to engage in favorable electrostatic interactions with the syn‐1,3‐diaxial hydrogens. These transannular interactions could not, however, be characterized either by atoms‐in‐molecules (AIM) analysis, since no bond paths connecting the interacting F and H atoms were identified, or by noncovalent interaction (NCI) analysis. Even for X = CH_2_, such electronic effects appear sufficient to render the *eae* conformer highly populated. The effect observed for X = O is comparable to that for X = S. Consistently, the average natural atomic charges on the *α*‐hydrogens are +0.1978 for compound **1** and +0.2153 and +0.2140 for compounds **2** and **3**, respectively.

Because fluorine decreases the acidity of geminal hydrogens in cyclohexanes, leading to the blueshift and shortening of the C─H bond typically associated with improper hydrogen bonding [25, 26], it is worth investigating whether the same behavior also occurs in the systems studied herein. First, the charges of the axial α‐hydrogens in axial 4‐fluorocyclohexanone and related derivatives were compared with those in the *eae* conformers of **1**, **2**, and **3**. Second, the NPA charges at C2 and C6 were analyzed to determine whether these carbon atoms become more positively charged than the corresponding hydrogens, thereby evaluating whether gauche interactions between C2/C6 and F4 are more relevant than unconventional hydrogen bonds. Figure 2 clearly shows that the axial hydrogens in the trifluorinated derivatives are less positively charged than those in the monofluorinated compounds, consistent with the behavior observed for fluorinated cyclohexanes. Therefore, geminal fluorination decreases the acidity of these hydrogens. In contrast, C2 and C6 become positively charged upon fluorination, whereas they are highly negative in the monofluorinated counterparts. Accordingly, the strong preference for the *eae* stereoisomers cannot be attributed solely to unconventional hydrogen bonding, since these interactions are stronger in the monofluorinated analogs, but also to favorable gauche interactions between the negatively charged fluorine atom at C4 and the positively charged α‐carbon atoms.

**FIGURE 2:**
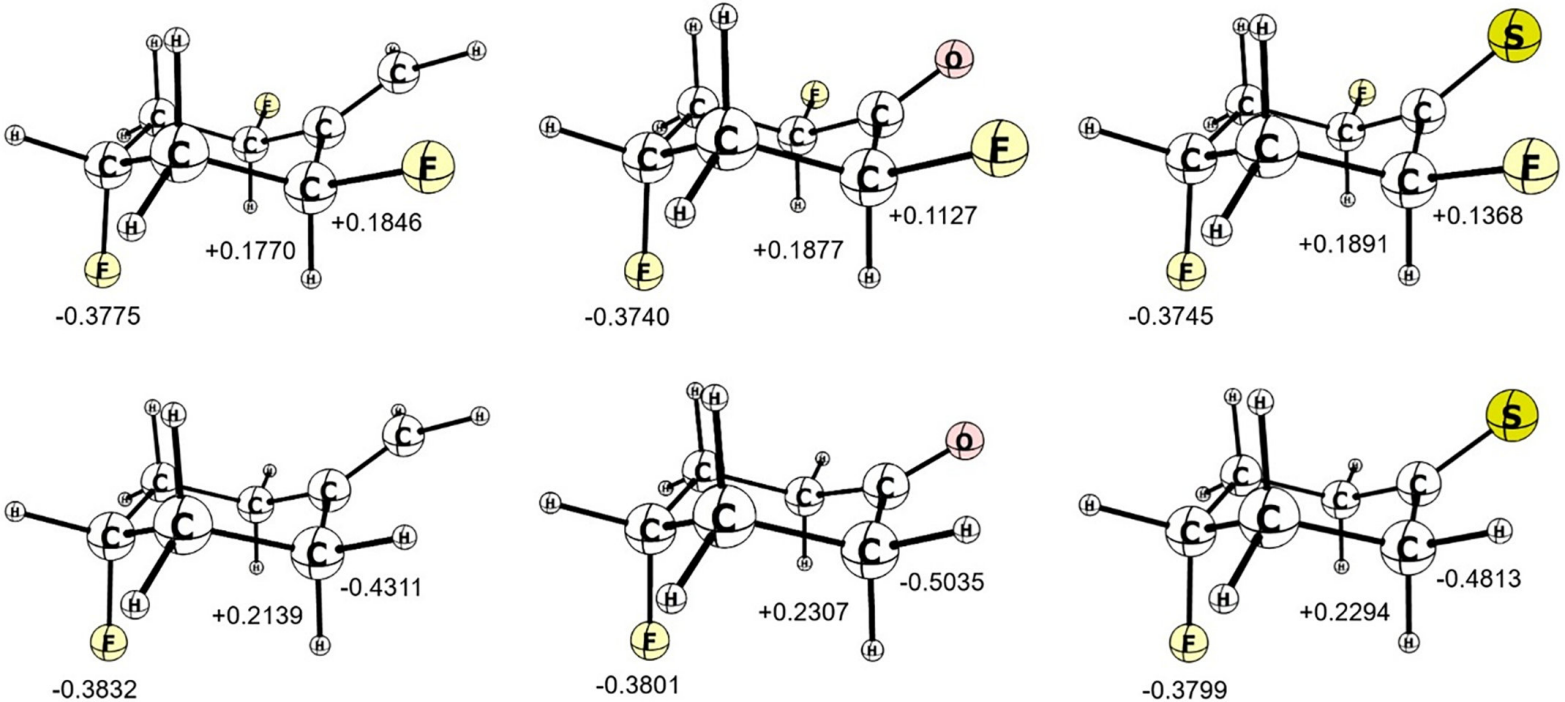
NPA charges on F4, the axial α‐hydrogens, and the α‐carbon atoms in **1** *eae*, **2** *eae*, and **3** *eae*, compared with those of their corresponding monofluorinated counterparts.

Although differences in atomic charges help rationalize the greater preference for the *eae* conformer in compounds **2** and **3** relative to **1**, a more quantitative evaluation of electrostatic stabilization can be achieved through natural Coulomb electrostatics (NCE) analysis within the natural bond orbital (NBO) framework (Table 1). For compounds **1** and **3**, the *eae* stereoisomer is electrostatically favored, largely due to stabilizing C4─F···H─C and gauche F4···C interactions (Figure 3). In contrast, compound **2** shows an electrostatic preference for the *aea* stereoisomer, followed by *aaa*. This behavior can be attributed to the equatorial α‐carbonyl fluorine atoms in *eae*, whose C─F bonds are nearly aligned with the polar C═O bond, leading to increased dipolar repulsion (Figure 4). Nevertheless, the *eae* stereoisomer remains the most stable form of compound **2**, a result that can be understood by considering the Lewis (*E*_L_) and non‐Lewis (*E*_NL_) contributions to the total electronic energies of the stereoisomers.

**FIGURE 3:**
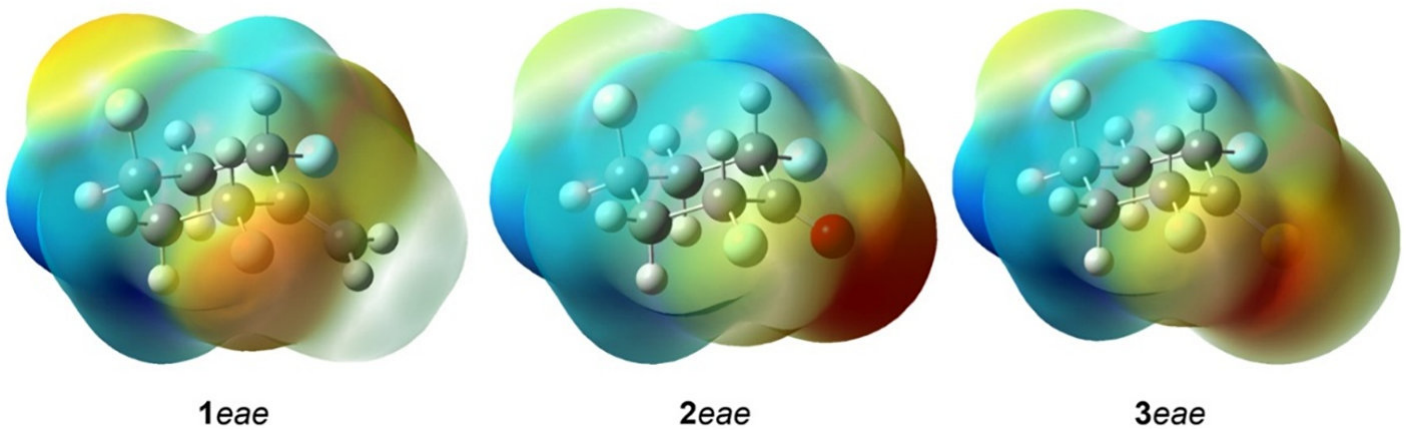
Electrostatic potential maps illustrating C─F···H─C contacts in **1** *eae*, **2** *eae*, and **3** *eae*. Blue regions correspond to more positive potentials, whereas red regions indicate more negative areas.

**FIGURE 4:**
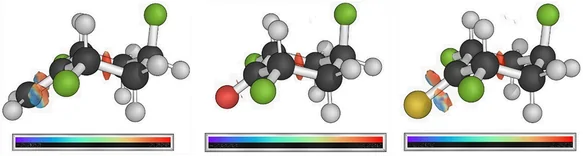
NCI plots for **1** *eae*, **2** *eae*, and **3** *eae* showing two interaction regions between the equatorial C─F bonds and the C═X group. These regions are predominantly blue (attractive) for X = CH_2_ and S, whereas they are smaller and red (repulsive) for X = O.

The electronic energy can be partitioned into Lewis and non‐Lewis contributions (*E* = *E*_L_ + *E*_NL_), where the former reflects 4‐electron/2‐orbital interactions largely associated with steric effects, and the latter accounts for stabilization arising from electron delocalization. The preference for the **2** *eae* stereoisomer results from a balance between strong hyperconjugative stabilization and moderate electrostatic attraction. Although the equatorial fluorine atoms at C2 and C6 introduce repulsive interactions with the carbonyl group, the corresponding axial hydrogens enable significant *σ*_C─H_ → *π* *_C═O_ interactions, amounting to 5.37 kcal mol^−1^ each. In contrast, for **2** *aaa* and **2** *aea*, the analogous *σ*_C─F_ → π*_C═O_ and *n*_F_ *→* π*_C═O_ interactions are considerably weaker, with total contributions of only 2.47 and 2.58 kcal mol^−1^, respectively.

In water, a polar solvent that stabilizes dipolar species, the relative energetics change, particularly for compound **1**. Although the **1** *eae* stereoisomer remains the most favored from an electrostatic standpoint, **1** *aea* becomes the most stable overall. This shift arises from its higher polarity, making its energy more sensitive to solvation effects. An even more pronounced case is observed for the highly polar **1** *aaa* stereoisomer, which is by far the least stable in the gas phase but becomes much closer in energy in an aqueous solution, with a difference reduced to only 0.7 kcal mol^−1^.

The behavior of compound **1** contrasts with that of **2** and **3** because, in **1**, the C─F bonds are the sole contributors to molecular polarity, whereas in **2** and **3** the C═O and C═S groups also contribute significantly to the overall dipole moment. As a result, the *eae* stereoisomer in **2** and **3** remains highly polar and continues to be the most stable form in solution. Since the NCE energies in implicit water follow the same trends observed in the gas phase, this suggests that the unconventional C─F···H─C hydrogen bonding, as well as the electrostatic gauche interactions, is not significantly affected by the change in medium. It should be noted, however, that implicit solvation accounts only for dielectric effects and does not capture specific solute–solvent interactions.

## 3 Conclusion

The unconventional C─F···H─C hydrogen bond plays a significant role in stabilizing an axial fluorine atom at C4 in methylidenecyclohexanes, cyclohexanones, and cyclohexanethiones. This effect is further reinforced by electrostatic F4···C interactions associated with the gauche effect because α‐fluorination renders the α‐carbon atoms more positively charged. In compounds **1** and **3**, the stability of the *eae* stereoisomer is therefore largely governed by this electrostatic contribution. In contrast, in compound **2**, the equatorial orientation of the α‐fluorine atoms in **2** *eae* gives rise to pronounced dipolar repulsion with the nearly aligned C═O bond. Consequently, the preference for **2** *eae* is driven mainly by stabilizing *σ*_C─H_ → π*_C═O_ hyperconjugative interactions rather than by electrostatic effects.

In implicit aqueous solution, both the C─F···H─C interaction and the electrostatic gauche effect remain operative. However, solvent polarity substantially modifies the relative conformational energies by preferentially stabilizing the more polar stereoisomers. These findings emphasize that the conformational effects of fluorination cannot be attributed to a single stereoelectronic factor. Rather, the balance between electrostatic interactions, unconventional hydrogen bonding, and hyperconjugation depends on the molecular framework, the orientation of the fluorine substituents, and the nature of the interacting functional groups.

This interplay is expected to be relevant to other fluorinated molecular systems in which C─F bonds are positioned near polarized C─H bonds, carbonyl or other π‐accepting groups, or electrostatically complementary molecular regions. In such systems, fluorination may simultaneously modify local charge distributions, dipole moments, and the availability of donor and acceptor orbitals. The present results therefore suggest that conformational analyses of fluorinated compounds should consider electrostatic and hyperconjugative contributions together, rather than interpreting fluorine‐dependent preferences exclusively in terms of hydrogen bonding, the gauche effect, or any other isolated interaction.

## 4 Computational Methods

The geometries of the compounds shown in Figure 1 were fully optimized, and harmonic frequency calculations were performed to obtain standard Gibbs free energies at the B3LYP/def2‐TZVP level of density functional theory [27, 28, 29], which has previously been shown to provide conformational energies in good agreement with CCSD(T) results [30]. All calculations were carried out using the Gaussian 16 program [31]. This agreement was further verified through single‐point energy refinements at the DLPNO‐CCSD(T)/CBS level (Supporting Information) [32, 33]. The optimized structures were subsequently subjected to NBO analyses at the same level of theory using the NBO 7 program [34]. The NBODEL and NCE keywords were employed to partition the electronic energies into Lewis and non‐Lewis contributions and to obtain NCE energies, respectively. These analyses were performed with the ORCA 6.0 program [35].

## Funding

This work was supported by the Coordenação de Aperfeiçoamento de Pessoal de Nível Superior (001), Conselho Nacional de Desenvolvimento Científico e Tecnológico (306830/2021‐3), and Fundação de Amparo à Pesquisa do Estado de Minas Gerais (n. a.).

## Conflicts of Interest

The author declares no conflicts of interest.

## Supporting information

Standard coordinates and absolute energies for the optimized geometries.

## Data Availability

The data that support the findings of this study are available in the Supporting Information of this article.
