# Supplementary material for: Electrostatic Effects on the Stability of 2,4,6‐Trifluoromethylidenecyclohexanes, Cyclohexanones, and Cyclohexanethiones: Beyond Nonconventional Hydrogen Bonding
Source: Chemphyschem. 2026 Oct 29;27(21):e70619. doi: 10.1002/cphc.70619 (PMC13719128; doi:10.1002/cphc.70619)
Supplement: Supplementary file 1 [file CPHC-27-e70619-s001.pdf]

SUPPORTING INFORMATION

Electrostatic Effects on the Stability of 2,4,6-Trifluoromethylidenecyclohexanes, Cyclohexanones, and Cyclohexanethiones: Beyond Nonconventional Hydrogen Bonding

Matheus P. Freitas[a]*

[a] Department of Chemistry, Institute of Natural Sciences, Federal University of Lavras, 37200-900, Lavras, MG, Brazil

* E-mail: matheus@ufla.br

Pages S2-S25: Cartesian coordinates and absolute energies (in hartrees) of the optimized geometries for trifluoromethylidenecyclohexanes, trifluorocyclohexanones, and trifluorocyclohexanethiones, calculated at the B3LYP/def2-TZVP level in both the gas phase and implicit water (SMD).

1*aaa* (gas)

6	-1.572863000	0.000186000	-0.687004000
6	-0.750209000	1.272945000	-0.847369000
6	0.561192000	1.283583000	-0.067443000
6	0.560729000	-1.283716000	-0.067160000
6	-0.750500000	-1.272737000	-0.847559000
1	-0.511340000	1.391660000	-1.909001000
1	-1.351818000	2.134193000	-0.551495000
1	-0.511542000	-1.391250000	-1.909200000
1	-1.352291000	-2.133898000	-0.551882000
6	1.333681000	-0.000251000	-0.228664000
9	0.286215000	1.497776000	1.290705000
9	0.284829000	-1.497444000	1.291030000
1	1.176856000	2.127079000	-0.389224000
1	1.176121000	-2.127555000	-0.388378000
1	-2.383973000	0.000259000	-1.421041000
9	-2.192960000	0.000267000	0.566672000
6	2.620242000	-0.000688000	-0.558270000
1	3.171116000	0.921925000	-0.697546000
1	3.170490000	-0.923738000	-0.697078000

G^0 = -571.78662 hartrees, E (DLPNO-CCSD(T)/CBS) = -571.1235933 hartrees

1*eee* (gas)

6	1.690445000	0.000012000	0.254083000
6	0.940321000	-1.263198000	-0.141036000
6	-0.468587000	-1.253002000	0.449294000
6	-0.469300000	1.252933000	0.449673000
6	0.939877000	1.263166000	-0.140319000
1	0.863181000	-1.307810000	-1.229978000
1	1.480802000	-2.146879000	0.200584000
1	-0.414724000	1.332598000	1.543048000
1	0.862968000	1.308431000	-1.229244000
1	1.480515000	2.146655000	0.201704000
1	-0.413651000	-1.331745000	1.542839000
6	-1.217563000	-0.000195000	0.071710000
9	-1.146238000	-2.389398000	0.016918000
9	-1.147156000	2.389086000	0.016386000
9	2.940177000	0.000745000	-0.374602000
1	1.895249000	-0.000390000	1.330254000
6	-2.386211000	-0.000358000	-0.552896000
1	-2.874369000	-0.928060000	-0.815501000
1	-2.874919000	0.927162000	-0.815076000

G^0 = -571.79174 hartrees, E (DLPNO-CCSD(T)/CBS) = -571.1294513 hartrees

1*aea* (gas)

6	-1.537040000	0.000112000	0.101838000
6	-0.864502000	1.260052000	-0.418582000
6	0.621034000	1.283450000	-0.078028000
6	0.620747000	-1.283544000	-0.077677000
6	-0.864729000	-1.259788000	-0.419019000
1	-0.977790000	1.297576000	-1.506170000
1	-1.344436000	2.147234000	-0.002634000
1	-0.977837000	-1.296898000	-1.506634000
1	-1.344995000	-2.146936000	-0.003419000
6	1.316719000	-0.000190000	-0.453235000
9	-2.875030000	0.000258000	-0.323450000
1	-1.558124000	-0.000065000	1.192577000
9	0.748919000	1.492694000	1.309466000
9	0.747524000	-1.492475000	1.310067000
1	1.112175000	2.131018000	-0.559516000
1	1.111973000	-2.131348000	-0.558553000
6	2.457204000	-0.000622000	-1.133736000
1	2.945200000	0.921745000	-1.425049000
1	2.944512000	-0.923449000	-1.424721000

G^0 = -571.79194 hartrees, E (DLPNO-CCSD(T)/CBS) = -571.1294225 hartrees

1*eae* (gas)

6	-1.741589000	-0.000089000	0.466965000
6	-0.914810000	-1.270405000	0.592176000
6	0.294004000	-1.250916000	-0.341519000
6	0.293890000	1.250869000	-0.341509000
6	-0.914711000	1.270106000	0.592594000
1	-0.554610000	-1.358809000	1.620656000
1	-1.539070000	-2.138914000	0.377903000
1	-2.551163000	-0.000138000	1.199284000
1	-0.043354000	1.321128000	-1.381001000
1	-0.554153000	1.357969000	1.620992000
1	-1.538979000	2.138781000	0.379001000
1	-0.042937000	-1.321115000	-1.381152000
6	1.112128000	0.000029000	-0.152886000
9	1.060231000	-2.390396000	-0.100671000
9	1.060025000	2.390531000	-0.100792000
9	-2.363632000	0.000147000	-0.801063000
6	2.395853000	0.000117000	0.177527000
1	2.933112000	-0.927627000	0.313399000
1	2.932950000	0.927921000	0.313570000

G^0 = -571.79426 hartrees, E (DLPNO-CCSD(T)/CBS) = -571.1322751 hartrees

2*aaa* (gas)

6	-1.504087000	0.000002000	-0.727407000
6	-0.679334000	1.279732000	-0.848865000
6	0.591995000	1.296148000	-0.001749000
6	0.591987000	-1.296151000	-0.001743000
6	-0.679340000	-1.279731000	-0.848863000
1	-0.386964000	1.403922000	-1.896481000
1	-1.297573000	2.138962000	-0.581826000
1	-0.386965000	-1.403920000	-1.896479000
1	-1.297583000	-2.138959000	-0.581828000
6	1.370623000	-0.000005000	-0.233455000
8	2.475515000	-0.000010000	-0.713971000
9	0.270086000	1.418464000	1.346223000
9	0.270071000	-1.418457000	1.346228000
1	1.235348000	2.136738000	-0.266264000
1	1.235334000	-2.136747000	-0.266250000
1	-2.276959000	0.000005000	-1.501171000
9	-2.182360000	0.000005000	0.493611000

G^0 = -607.74471 hartrees, E (DLPNO-CCSD(T)/CBS) = -607.0390228 hartrees

2*eee* (gas)

6	1.662445000	-0.000087000	0.276758000
6	0.922935000	-1.264641000	-0.139127000
6	-0.496494000	-1.274725000	0.428336000
6	-0.496440000	1.274758000	0.428366000
6	0.923056000	1.264609000	-0.138924000
1	0.866429000	-1.304032000	-1.230019000
1	1.461073000	-2.149350000	0.202578000
1	-0.468672000	1.327840000	1.524615000
1	0.866644000	1.304205000	-1.229814000
1	1.461256000	2.149202000	0.202982000
1	-0.468557000	-1.327755000	1.524584000
6	-1.246310000	0.000035000	0.030793000
8	-2.291918000	0.000064000	-0.552573000
9	-1.174593000	-2.386661000	-0.018873000
9	-1.174411000	2.386725000	-0.018989000
9	2.922577000	-0.000078000	-0.323187000
1	1.839873000	-0.000192000	1.357886000

G^0 = -607.74968 hartrees, E (DLPNO-CCSD(T)/CBS) = -607.0435755 hartrees

2*aea* (gas)

6	-1.497315000	-0.000006000	0.142307000
6	-0.835219000	1.267537000	-0.385838000
6	0.654339000	1.298764000	-0.065238000
6	0.654362000	-1.298749000	-0.065279000
6	-0.835205000	-1.267552000	-0.385811000
1	-0.963582000	1.310539000	-1.471878000
1	-1.313463000	2.152029000	0.038410000
1	-0.963616000	-1.310596000	-1.471843000
1	-1.313413000	-2.152038000	0.038489000
6	1.313824000	0.000020000	-0.542288000
8	2.208629000	0.000042000	-1.347319000
9	-2.836685000	-0.000022000	-0.266196000
1	-1.503135000	0.000010000	1.233113000
9	0.826012000	1.422278000	1.316510000
9	0.826103000	-1.422300000	1.316466000
1	1.160291000	2.140688000	-0.537903000
1	1.160312000	-2.140650000	-0.537987000

G^0 = -607.7491 hartrees, E (DLPNO-CCSD(T)/CBS) = -607.0437956 hartrees

2*eae* (gas)

6	-1.724227000	0.000988000	0.446104000
6	-0.901965000	-1.269655000	0.595871000
6	0.316917000	-1.271929000	-0.329470000
6	0.318399000	1.271583000	-0.329522000
6	-0.900551000	1.270735000	0.595726000
1	-0.554286000	-1.349202000	1.629575000
1	-1.522130000	-2.141842000	0.385931000
1	-2.550569000	0.001501000	1.159295000
1	-0.005236000	1.318275000	-1.374942000
1	-0.552820000	1.350031000	1.629433000
1	-1.519771000	2.143566000	0.385671000
1	-0.006896000	-1.318212000	-1.374854000
6	1.143359000	-0.000653000	-0.131073000
8	2.300731000	-0.001319000	0.179942000
9	1.086908000	-2.388471000	-0.080048000
9	1.089655000	2.387232000	-0.079986000
9	-2.310534000	0.001241000	-0.836129000

G^0 = -607.75297 hartrees, E (DLPNO-CCSD(T)/CBS) = -607.0472314 hartrees

3*aaa* (gas)

6	-1.700015000	-0.000001000	-0.895961000
6	-0.863762000	1.275341000	-0.889563000
6	0.278019000	1.280279000	0.128210000
6	0.278020000	-1.280278000	0.128209000
6	-0.863763000	-1.275344000	-0.889560000
1	-0.428984000	1.410450000	-1.884407000
1	-1.508934000	2.134557000	-0.696792000
1	-0.428987000	-1.410456000	-1.884405000
1	-1.508934000	-2.134560000	-0.696786000
6	1.083908000	0.000000000	0.017141000
9	-0.240993000	1.398042000	1.416929000
9	-0.240990000	-1.398041000	1.416931000
1	0.932367000	2.136999000	-0.032266000
1	0.932369000	-2.136998000	-0.032266000
1	-2.346729000	-0.000001000	-1.777957000
9	-2.556803000	0.000001000	0.207664000
16	2.652029000	0.000001000	-0.372481000

G^0 = -930.7001 hartrees, E (DLPNO-CCSD(T)/CBS) = -929.6304711 hartrees

3*eee* (gas)

6	-1.965564000	0.000007000	0.186145000
6	-1.182654000	1.261294000	-0.143860000
6	0.183357000	1.247882000	0.547577000
6	0.183356000	-1.247883000	0.547582000
6	-1.182664000	-1.261293000	-0.143835000
1	-1.027837000	1.318708000	-1.223870000
1	-1.735659000	2.147965000	0.167544000
1	0.050980000	-1.259068000	1.638146000
1	-1.027859000	-1.318733000	-1.223845000
1	-1.735675000	-2.147951000	0.167594000
1	0.050958000	1.259061000	1.638139000
6	0.963025000	-0.000001000	0.181851000
9	0.865109000	2.399883000	0.220701000
9	0.865100000	-2.399886000	0.220686000
9	-3.153179000	0.000002000	-0.549245000
1	-2.261622000	0.000021000	1.240639000
16	2.406269000	-0.000003000	-0.530399000

G^0 = -930.70483 hartrees, E (DLPNO-CCSD(T)/CBS) = -929.634678 hartrees

3*aea* (gas)

6	-1.794994000	-0.000002000	-0.097366000
6	-1.025871000	1.262791000	-0.458050000
6	0.356850000	1.282679000	0.187988000
6	0.356846000	-1.282676000	0.188010000
6	-1.025870000	-1.262798000	-0.458035000
1	-0.910379000	1.312897000	-1.544539000
1	-1.576145000	2.150188000	-0.140930000
1	-0.910373000	-1.312914000	-1.544523000
1	-1.576146000	-2.150193000	-0.140910000
6	1.109884000	-0.000003000	-0.120224000
9	-3.004390000	-0.000006000	-0.805268000
1	-2.053476000	0.000004000	0.962248000
9	0.203302000	1.404832000	1.575347000
9	0.203288000	-1.404797000	1.575372000
1	0.940107000	2.139909000	-0.144744000
1	0.940104000	-2.139915000	-0.144697000
16	2.541590000	-0.000012000	-0.866557000

G^0 = -930.70456 hartrees, E (DLPNO-CCSD(T)/CBS) = -929.6353786 hartrees

3*eae* (gas)

6	1.979006000	-0.000020000	0.520665000
6	1.145123000	1.266487000	0.607163000
6	-0.024798000	1.245089000	-0.382618000
6	-0.024831000	-1.245096000	-0.382635000
6	1.145119000	-1.266526000	0.607108000
1	0.740300000	1.364765000	1.617771000
1	1.768965000	2.138750000	0.409265000
1	2.752052000	-0.000043000	1.291409000
1	0.361862000	-1.250416000	-1.408365000
1	0.740307000	-1.364853000	1.617717000
1	1.768961000	-2.138779000	0.409167000
1	0.361947000	1.250387000	-1.408329000
6	-0.864327000	0.000008000	-0.192438000
9	-0.762538000	2.399999000	-0.223929000
9	-0.762595000	-2.399989000	-0.223905000
9	2.656962000	0.000007000	-0.716099000
16	-2.425789000	0.000024000	0.205206000

G^0 = -930.7082 hartrees, E (DLPNO-CCSD(T)/CBS) = -929.638362 hartrees

1*aaa* (implicit water)

6	-1.568444000	0.000087000	-0.695495000
6	-0.752026000	1.269924000	-0.856916000
6	0.560062000	1.281046000	-0.090191000
6	0.559408000	-1.281387000	-0.089723000
6	-0.752513000	-1.270121000	-0.856638000
1	-0.512378000	1.374288000	-1.918803000
1	-1.350102000	2.138956000	-0.577170000
1	-0.512617000	-1.374505000	-1.918476000
1	-1.350848000	-2.138977000	-0.576976000
6	1.332871000	-0.000396000	-0.230928000
9	0.278721000	1.495240000	1.295509000
9	0.277711000	-1.494315000	1.296168000
1	1.172588000	2.128696000	-0.394378000
1	1.171568000	-2.129570000	-0.393144000
1	-2.396992000	0.000229000	-1.404005000
9	-2.179502000	0.000378000	0.591655000
6	2.626863000	-0.000693000	-0.534714000
1	3.174749000	0.924685000	-0.669635000
1	3.174340000	-0.926292000	-0.669779000

G^0 = -571.80003 hartrees, E (DLPNO-CCSD(T)/CBS) = -571.137038 hartrees

1*eee* (implicit water)

6	1.684579000	0.000430000	0.262612000
6	0.942259000	-1.262691000	-0.137962000
6	-0.463650000	-1.248387000	0.452545000
6	-0.464815000	1.248219000	0.452911000
6	0.941030000	1.262736000	-0.138188000
1	0.866725000	-1.303648000	-1.227487000
1	1.479247000	-2.143818000	0.214562000
1	-0.414536000	1.333879000	1.542460000
1	0.865084000	1.303170000	-1.227694000
1	1.477936000	2.144290000	0.213561000
1	-0.413821000	-1.333058000	1.542331000
6	-1.217521000	-0.000391000	0.073906000
9	-1.147237000	-2.392143000	0.006887000
9	-1.149199000	2.391512000	0.006795000
9	2.944240000	0.001299000	-0.381472000
1	1.898358000	0.000538000	1.333167000
6	-2.390989000	-0.000805000	-0.544156000
1	-2.886695000	-0.925697000	-0.805808000
1	-2.887890000	0.923665000	-0.804993000

G^0 = -571.79918 hartrees, E (DLPNO-CCSD(T)/CBS) = -571.1366822 hartrees

1*aea* (implicit water)

6	-1.534522000	0.000051000	0.109360000
6	-0.868616000	1.260876000	-0.409887000
6	0.617921000	1.280847000	-0.092309000
6	0.617558000	-1.281052000	-0.091946000
6	-0.868901000	-1.260983000	-0.409795000
1	-0.980995000	1.299939000	-1.496897000
1	-1.341673000	2.149320000	0.011107000
1	-0.981044000	-1.299975000	-1.496826000
1	-1.342212000	-2.149316000	0.011124000
6	1.312332000	-0.000250000	-0.462425000
9	-2.885377000	0.000258000	-0.325953000
1	-1.579743000	0.000073000	1.198558000
9	0.763400000	1.487464000	1.316820000
9	0.762682000	-1.486852000	1.317368000
1	1.105255000	2.131869000	-0.564588000
1	1.104887000	-2.132418000	-0.563599000
6	2.453891000	-0.000501000	-1.142899000
1	2.935760000	0.924642000	-1.436906000
1	2.935446000	-0.925882000	-1.436685000

G^0 = -571.80119 hartrees, E (DLPNO-CCSD(T)/CBS) = -571.1382206 hartrees

1*eae* (implicit water)

6	-1.738566000	-0.000272000	0.475522000
6	-0.914125000	-1.270124000	0.585114000
6	0.293399000	-1.244966000	-0.345119000
6	0.293008000	1.244962000	-0.345072000
6	-0.914282000	1.269617000	0.585630000
1	-0.561063000	-1.356428000	1.615791000
1	-1.539128000	-2.136279000	0.364862000
1	-2.535222000	-0.000423000	1.218255000
1	-0.029987000	1.317786000	-1.386995000
1	-0.560818000	1.355285000	1.616223000
1	-1.539414000	2.135878000	0.366179000
1	-0.029218000	-1.317905000	-1.387173000
6	1.116812000	0.000149000	-0.152813000
9	1.068035000	-2.393336000	-0.100426000
9	1.067264000	2.393720000	-0.100620000
9	-2.393046000	-0.000080000	-0.791677000
6	2.401377000	0.000371000	0.178724000
1	2.944631000	-0.924349000	0.317525000
1	2.944198000	0.925276000	0.317925000

G^0 = -571.80067 hartrees, E (DLPNO-CCSD(T)/CBS) = -571.1380932 hartrees

2*aaa* (implicit water)

6	-1.523399000	0.000001000	-0.706827000
6	-0.707173000	1.274156000	-0.856160000
6	0.581083000	1.297164000	-0.048791000
6	0.581084000	-1.297164000	-0.048792000
6	-0.707172000	-1.274153000	-0.856162000
1	-0.436146000	1.374948000	-1.910871000
1	-1.310096000	2.145579000	-0.594716000
1	-0.436143000	-1.374942000	-1.910873000
1	-1.310096000	-2.145578000	-0.594722000
6	1.361027000	0.000001000	-0.252110000
8	2.501521000	0.000001000	-0.656796000
9	0.292976000	1.423817000	1.325745000
9	0.292975000	-1.423821000	1.325742000
1	1.211863000	2.141773000	-0.320785000
1	1.211864000	-2.141771000	-0.320789000
1	-2.334880000	0.000001000	-1.434401000
9	-2.154976000	-0.000001000	0.565687000

G^0 = -607.76043 hartrees, E (DLPNO-CCSD(T)/CBS) = -607.0540587 hartrees

2*eee* (implicit water)

6	1.657910000	-0.000053000	0.287470000
6	0.926949000	-1.264732000	-0.136826000
6	-0.489276000	-1.268649000	0.430362000
6	-0.489243000	1.268670000	0.430385000
6	0.927020000	1.264711000	-0.136699000
1	0.871143000	-1.297168000	-1.227836000
1	1.458398000	-2.147590000	0.217821000
1	-0.470323000	1.335242000	1.522642000
1	0.871264000	1.297269000	-1.227708000
1	1.458511000	2.147498000	0.218063000
1	-0.470245000	-1.335186000	1.522621000
6	-1.231274000	0.000020000	0.045911000
8	-2.295706000	0.000039000	-0.526231000
9	-1.186582000	-2.376934000	-0.042191000
9	-1.186472000	2.376972000	-0.042264000
9	2.927717000	-0.000044000	-0.326932000
1	1.842410000	-0.000119000	1.363105000

G^0 = -607.76832 hartrees, E (DLPNO-CCSD(T)/CBS) = -607.0617103 hartrees

2*aea* (implicit water)

6	-1.502567000	0.000011000	0.154538000
6	-0.850903000	1.265967000	-0.380981000
6	0.639477000	1.298970000	-0.091012000
6	0.639436000	-1.298995000	-0.090966000
6	-0.850933000	-1.265953000	-0.381003000
1	-0.984006000	1.304607000	-1.465940000
1	-1.315619000	2.154779000	0.048436000
1	-0.983993000	-1.304553000	-1.465968000
1	-1.315693000	-2.154762000	0.048371000
6	1.307344000	-0.000032000	-0.546045000
8	2.253705000	-0.000059000	-1.298529000
9	-2.856986000	0.000034000	-0.252476000
1	-1.521428000	-0.000001000	1.244274000
9	0.847267000	1.423374000	1.299875000
9	0.847147000	-1.423335000	1.299935000
1	1.131580000	2.144693000	-0.567122000
1	1.131537000	-2.144757000	-0.567008000

G^0 = -607.76073 hartrees, E (DLPNO-CCSD(T)/CBS) = -607.0544149 hartrees

2*eae* (implicit water)

6	-1.725742000	-0.000023000	0.448993000
6	-0.905607000	-1.269906000	0.586670000
6	0.316680000	-1.264681000	-0.329354000
6	0.316645000	1.264689000	-0.329353000
6	-0.905640000	1.269882000	0.586672000
1	-0.567710000	-1.344844000	1.623010000
1	-1.522601000	-2.140661000	0.365351000
1	-2.538092000	-0.000034000	1.174325000
1	0.016414000	1.324646000	-1.379183000
1	-0.567748000	1.344827000	1.623014000
1	-1.522655000	2.140622000	0.365354000
1	0.016450000	-1.324644000	-1.379184000
6	1.131979000	0.000015000	-0.139852000
8	2.304806000	0.000030000	0.155983000
9	1.109987000	-2.378211000	-0.060998000
9	1.109924000	2.378240000	-0.060999000
9	-2.344621000	-0.000029000	-0.831692000

G^0 = -607.77009 hartrees, E (DLPNO-CCSD(T)/CBS) = -607.0636484 hartrees

3*aaa* (implicit water)

6	-1.724749000	-0.000006000	-0.874332000
6	-0.892912000	1.270346000	-0.896923000
6	0.274863000	1.281061000	0.081086000
6	0.274860000	-1.281059000	0.081101000
6	-0.892910000	-1.270358000	-0.896911000
1	-0.481745000	1.384025000	-1.903389000
1	-1.522209000	2.141333000	-0.705647000
1	-0.481739000	-1.384037000	-1.903376000
1	-1.522203000	-2.141348000	-0.705636000
6	1.078944000	-0.000002000	-0.016385000
9	-0.211434000	1.403636000	1.403325000
9	-0.211447000	-1.403609000	1.403341000
1	0.914962000	2.143525000	-0.089500000
1	0.914957000	-2.143528000	-0.089463000
1	-2.416332000	-0.000008000	-1.716669000
9	-2.544432000	0.000001000	0.286551000
16	2.661972000	-0.000006000	-0.349443000

G^0 = -930.71402 hartrees, E (DLPNO-CCSD(T)/CBS) = -929.6441591 hartrees

3*eee* (implicit water)

6	-1.962337000	0.000006000	0.198989000
6	-1.188036000	1.260515000	-0.142900000
6	0.177811000	1.242894000	0.543379000
6	0.177811000	-1.242894000	0.543383000
6	-1.188043000	-1.260515000	-0.142877000
1	-1.038926000	1.312167000	-1.224078000
1	-1.734653000	2.145186000	0.183305000
1	0.055785000	-1.262098000	1.631700000
1	-1.038944000	-1.312189000	-1.224056000
1	-1.734666000	-2.145175000	0.183348000
1	0.055764000	1.262090000	1.631694000
6	0.955778000	0.000000000	0.184071000
9	0.869468000	2.399259000	0.196831000
9	0.869462000	-2.399260000	0.196818000
9	-3.162140000	0.000001000	-0.544684000
1	-2.258636000	0.000018000	1.249352000
16	2.416579000	-0.000002000	-0.511014000

G^0 = -930.72009 hartrees, E (DLPNO-CCSD(T)/CBS) = -929.6494622 hartrees

3*aea* (implicit water)

6	-1.803383000	0.000000000	-0.073955000
6	-1.045746000	1.261581000	-0.449394000
6	0.349147000	1.283256000	0.157290000
6	0.349147000	-1.283256000	0.157289000
6	-1.045746000	-1.261581000	-0.449394000
1	-0.941952000	1.307391000	-1.536807000
1	-1.583230000	2.152920000	-0.122208000
1	-0.941953000	-1.307391000	-1.536807000
1	-1.583230000	-2.152920000	-0.122207000
6	1.101814000	0.000000000	-0.144023000
9	-3.033204000	0.000000000	-0.774260000
1	-2.065618000	0.000000000	0.983738000
9	0.238112000	1.406800000	1.563747000
9	0.238113000	-1.406802000	1.563746000
1	0.915837000	2.145894000	-0.184070000
1	0.915838000	-2.145894000	-0.184072000
16	2.554108000	0.000001000	-0.853971000

G^0 = -930.71442 hartrees, E (DLPNO-CCSD(T)/CBS) = -929.6446469 hartrees

3*eae* (implicit water)

6	1.985473000	-0.000015000	0.514713000
6	1.155136000	1.265029000	0.599075000
6	-0.024883000	1.240152000	-0.375110000
6	-0.024909000	-1.240159000	-0.375123000
6	1.155141000	-1.265066000	0.599025000
1	0.767836000	1.356169000	1.616415000
1	1.774934000	2.136658000	0.388844000
1	2.753137000	-0.000028000	1.287434000
1	0.335115000	-1.255740000	-1.408406000
1	0.767871000	-1.356265000	1.616372000
1	1.774941000	-2.136680000	0.388735000
1	0.335178000	1.255713000	-1.408380000
6	-0.861151000	0.000005000	-0.189037000
9	-0.775597000	2.399320000	-0.196280000
9	-0.775645000	-2.399310000	-0.196260000
9	2.681116000	0.000011000	-0.726738000
16	-2.436670000	0.000019000	0.184452000

G^0 = -930.72199 hartrees, E (DLPNO-CCSD(T)/CBS) = -929.6515614 hartrees
